# Human African Trypanosomiasis in the Kafue National Park, Zambia

**DOI:** 10.1371/journal.pntd.0004567

**Published:** 2016-05-19

**Authors:** David Squarre, Ilunga Kabongo, Musso Munyeme, Chisoni Mumba, Wizaso Mwasinga, Lottie Hachaambwa, Chihiro Sugimoto, Boniface Namangala

**Affiliations:** 1 Zambia Wildlife Authority (ZAWA), Chilanga, Zambia; 2 Care for Business (CFB) Medical Center and Hospital, Lusaka, Zambia; 3 Department of Disease Control, School of Veterinary Medicine, University of Zambia, Lusaka, Zambia; 4 University Teaching Hospital (UTH), Lusaka, Zambia; 5 Research Centre for Zoonosis Control, Hokkaido University, Kita-Ku, Sapporo, Japan; 6 Department of Paraclinical Studies, School of Veterinary Medicine, University of Zambia, Lusaka, Zambia; Hospital Infantil de Mexico Federico Gomez, UNITED STATES

## Introduction

Human African Trypanosomiasis (HAT) is a neglected tropical disease [1] caused by *Trypanosoma brucei rhodesiense* (eastern and southern Africa) or *Trypanosoma brucei gambiense* (western and central Africa) and is transmitted through the bite of an infected tsetse fly (*Glossina* species) [2,3]. The tsetse flies acquire their infections from humans or animals harbouring the human pathogenic parasites [2]. The disease is endemic in tropical and subtropical Africa [4], where it affects low-income populations [3]. Whereas *T*. *b*. *rhodesiense* causes acute HAT [5,6], *T*. *b*. *gambiense* causes a more chronic form of the disease [5]. Although HAT has been re-emerging in most of the old foci within sub-Saharan Africa since the 1970s, with *T*. *b*. *gambiense* accounting for more than 98% of the reported cases [7], the latest World Health Organization (WHO) reports suggest that the number of new cases have been reduced [1]. In the year 2009, after continued control efforts, the number of cases of HAT reported dropped below 10,000 (9,878) for the first time in 50 years. This decline in number of cases has continued with 6,314 new cases reported in 2012 [1]. However, the estimated number of actual cases is about 20,000 and the estimated population at risk is 65 million people. Despite such progress, only a fraction of the population at risk for contracting HAT in sub-Saharan Africa is under surveillance and relatively few cases are diagnosed annually [8,9]. In particular, there is considerable underdiagnosis of *rhodesiense* HAT in sub-Saharan Africa, including Zambia, mainly due to lack of HAT surveillance and control programmes [10,11].

Historically, epidemics of *rhodesiense* HAT were reported from the northern and southern regions of the Luangwa Valley and the Kafue River Valley in the 1960s and early 1970s [12]. According to WHO [1], Zambia currently reports <100 new HAT cases annually, mainly from the old foci in the tsetse-infested Luangwa River Valley, including the Chama, Mpika, Chipata, Mambwe, and, recently, Rufunsa districts, where the disease is re-emerging [13–15].

The Kafue National Park (KNP) and its surrounding Game Management Areas (GMA) form the Kafue ecosystem, which is a vast and continuous wildlife conservation area located in the central part of Zambia [16] and rich in biodiversity of high biomass [17]. It is a pristine ecosystem that supports a wide variety of undisturbed flora and fauna of important conservation status [17]. The area also supports the communities that live there by harnessing the benefits from ecotourism and ecosystem services [18]. Importantly, it has abundant wildlife and tsetse flies [16].

The Kafue ecosystem has in the past reported cases and epidemics of HAT [16,19]. The Primitive Methodist Church of England established Nkala Mission in 1893, which was later abandoned in 1930 because of tsetse flies and sleeping sickness [16]. Today Nkala lies in the heart of the Kafue ecosystem. Another focus, Itumbi Safari Camp, which was opened in 1958 in the KNP, was closed down in 1959 due to severe cases of sleeping sickness [19]. This demonstrates the historical presence of HAT in the Kafue ecosystem. However, for over 50 years now no reports or notable incidences of HAT have been recorded in the area. Based on this fact, it has been assumed that the area was devoid of HAT despite the obvious presence of tsetse flies. The present report describes a case of HAT in an adult male from KNP, 16 km away from Itumbi Safari Camp, its diagnosis, and management.

## Case Presentation

A 47-year-old man was hospitalized at the Care for Business Medical Center and Hospital in Lusaka, Zambia, with initial complaints of frequent episodes of headache, fever, dizziness, body malaise, and erythematous skin rashes. Rapid diagnostic tests for malaria (Immuno Chromatographic Test and blood slide), typhoid (IgG/IgM), and tick fever (Weil Felix) were all negative. The history of the patient revealed that he owned a wildlife safari lodge in KNP, about 16 kilometres from the Itumbi Safari Camp.

The patient had just returned from the Park following heightened tourist peak visits around the festive holidays. Together with colleagues, he had been involved in a two-day pursuit of an injured wild animal that was wounded by poachers and needed clinical intervention. This pursuit led the team to be exposed to the deep forest and far-flung areas within KNP. The patient reported being bitten multiple times by tsetse flies and other biting arthropods.

**Fig 1:**
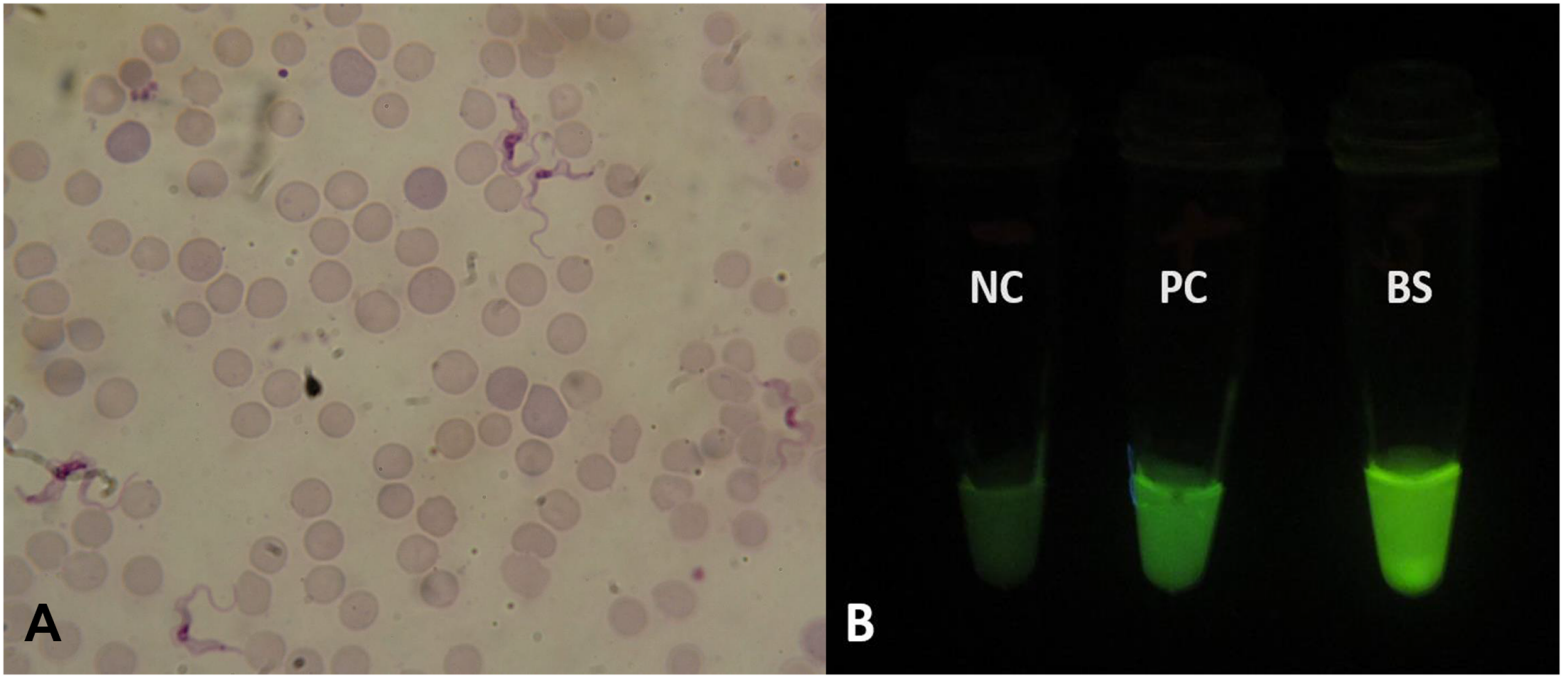
Microscopic and LAMP fluorescent visualisation of trypanosomes. Detection of trypanosomes in patient’s blood by (A) microscopy (×100 magnification) and (B) loop-mediated isothermal amplification (LAMP). Visual appearance of results for human serum resistance-associated gene (SRA)-LAMP of a patient with Early Stage human African trypanosomiasis (HAT). Loopamp Fluorescent detection reagent was added to the reaction mixture at the beginning of the assay. The reactions were incubated at 64°C for 30 minutes. In contrast to the light green background fluorescence in the negative samples, positive samples exhibit a bright fluorescent green colour when visualized under the transilluminator. NC: Negative control (distilled water); PC: Positive control (Trypanosoma brucei rhodesiense); BS: patient blood sample.

Following centrifugation of the patient’s blood, microscopic examination of Giemsa-stained thin buffy coat smears revealed the presence of several trypanosomes (Fig 1A), which, given the patient’s travel history, were thought to be *T*. *b*. *rhodesiense*. As shown in Fig 1B, loop-mediated isothermal amplification (LAMP) results confirmed the trypanosomes positive for the *T*. *b*. *rhodesiense*-specific human serum resistance-associated (SRA) gene. Blood test results were as follows: Alanine aminotransferase 114.3 U/L, bilirubin 82.7 μmol/L, creatinine 42.1 μmol/L, urea 2.78 mmol/L, leukocytes 4.8 × 109 cells/L, and platelets 18 × 10^9^/L.

Although the patient had a packed cell volume (PCV) value of 42% (reference 42% to 54%), he was critically ill. He was progressively getting weaker, having episodes of unconsciousness, and eventually became comatose. He was placed on assisted breathing ventilator as he could not breathe on his own. The presence of *T*. *b*. *rhodesiense*, which was only in the patient’s blood and not in the cerebrospinal fluid (CSF), coupled with ≤5 white blood cells (WBCs)/mm^3^ in the CSF, signified early-stage HAT, which was treated with Suramin [6] along with supportive therapy. A Suramin dose of 1 gram (g) dissolved in 5 centimetre cubed (cm^3^) of sterile water was administered slowly and intravenously on days 1, 3, 5, 14, and 21. The patient showed a tremendous improvement on the second day by returning to normal consciousness and was thus removed from the ventilator. He appeared brighter and more alert and was responsive to treatment. Before and after each treatment, a sample of blood was collected. Thin buffy coat slides were regularly examined by microscopy to track the course of the disease. His blood was cleared of all parasites after the fifth day of treatment and all clinical signs receded. The patient was hospitalized for observation until all treatment was completed. Other people that were in his company during his stay in the area were screened using microscopy and LAMP and were all negative.

## Case Discussion

Despite KNP having historical presence of HAT [16,17], no new cases were recorded for more than 50 years. This may be attributed to several reasons, including non-surveillance of HAT in the area and undetected HAT mortalities through misdiagnosis with other febrile conditions, such as malaria, tuberculosis, and HIV/AIDS [13,9]. Although most cases of re-emerging HAT in Zambia are mainly reported from Luangwa River Valley and, to a lesser extent, Zambezi River Valley [9,14,15,20,21], the patient described herein had no travel history to any of those foci, but was only bitten by tsetse flies from within KNP, strongly suggesting that he contracted the disease from that area. The operational area of the patient is just 16 kilometres from the Itumbi Safari Camp (old focus), which was closed down in 1959 due to severe cases of HAT [19]. This further highlights the continuous risk for park rangers, hunters, tourists, tourism facility operators, and the surrounding population who may become infected, as wildlife in this protected area are niches for HAT [23]. The infections at this stage are probably the result of an ecological disturbance that forces an encounter between an infected fly and humans.

The occurrence and distribution of tsetse fly in the Kafue ecosystem form a patchy mosaic of areas with varying degrees of fly densities ranging from nil/low to some with very high densities [16]. The area provides a suitable habitat and competent wildlife reservoir that support a thriving vector population. The main objective of protecting this conservation area is to preserve its wildlife and biodiversity by limiting and controlling the anthropogenic activity that directly or indirectly affects and threatens the biodiversity integrity [17]. Tsetse flies are also protected as part of this biodiversity despite being known vectors that transmit HAT. Arguably, HAT, more than any other disease, has been intertwined with the conservation of biodiversity [22]. By reason of the fly being protected in conservation areas and its habitat being adequately preserved and undisturbed, tsetse flies have flourished and further maintained the circulation of the parasite(s) they transmit. It can also be said that biodiversity protection, to an extent, reduces the ease of spread of HAT by hindering the effortless encounter of humans with high tsetse fly-infested areas and thus prevents disease transmission.

Human clinical case diagnosis should start with the recognition of the endemic presence of the disease in the area or ecoregion. Through this case, the presence of HAT in the area has been demonstrated. It is therefore recommended that all febrile conditions with a clinical picture resembling septicaemia or malaria should have HAT on top of its differential diagnostic list. This is important because early detection and treatment is key to case management [13]. HAT progresses through distinct clinical stages that invariably lead to death if left untreated [1]. In the present case, although there was no invasion of the central nervous system, the reported HAT was diagnosed late in light of the rapid progression of the disease due to absence of historical incidence or recognized/established presence of the disease in the area. This is further demonstrated by the lack of therapeutic drugs and incidences recorded from the local health care facility. It was, however, diagnosed by considering travel history and recollected multiple tsetse fly bites. This report further underscores the importance of accurate diagnosis in the management of HAT. Thus, although the patient had deteriorated to a comatose state with assisted ventilation, Therapeutic intervention with Suramin provided a complete cure of the disease.

It is important to not only reinforce local health care facilities in areas with demonstrable risk of HAT with relatively quick diagnostic tools such as LAMP and microscopy in addition to therapeutic drugs to avert non-detected HAT-related deaths but also provide the correct and timely treatment of diagnosed cases. Mitigation of HAT in Zambia has primarily occurred through passive detection and treatment [13]. Control of HAT requires a multi-sectorial approach by establishing effective coordination of various effective strategies with wildlife managers/ecologists, tsetse biologists, medics, veterinarians, and the media [19] in a model of the one health approach.

HAT is re-emerging in Zambia’s old foci, mainly in Luangwa and, to a lesser extent, Zambezi River Valleys, as is the case with other sub-Saharan African countries. The present article reports on a HAT case from KNP, about 50 years after the last case of the disease was documented in that old HAT focus. This report is a further reminder for the need for continuous surveillance of HAT. We envisage that this report will stimulate further research to investigate the prevalence of the human-infective trypanosome species in tsetse flies and possibly wildlife from KNP using user-friendly, specific, and sensitive tests such as LAMP to determine the associated risks of contracting HAT by the local inhabitants, park rangers, tourists, and hunters. Furthermore, we recommend the use of micro-satellite analysis to determine the relatedness of the *T*. *b*. *rhodesiense* species from KNP to those from other HAT foci in Zambia where re-emerging cases of the disease are being reported.

**Key Learning Points**

- This report demonstrates the presence and resurgence of HAT in an old focus after 50 years of no reports of cases. This is the first reported case of HAT from the Kafue National Park (KNP) of Zambia after more than 50 years since the last documented case. This case demonstrates the presence of HAT and suggests that HAT may be re-emerging in that old focus.
- Clinicians in local health centres should include HAT on top of differential diagnosis for all febrile conditions resembling malaria, septicaemia, tick fever, and other similar clinical ailments. We further highlight the need for continuous surveillance of HAT in the area.
- This report highlights HAT as a health problem in the area and emphasizes the use of user-friendly diagnostic tools such as LAMP and microscopy to avert non-detected HAT-related deaths and further stock up HAT drugs for early treatment and management.
- Raise awareness to the public (communities, tourists, and game rangers) on the risk of infection of HAT in the area. The poor communities that live in the surrounding area, the large volume of international tourists that visit the area for wildlife and nature-based tourism, and the game rangers and tourist facility operators that work in the area are at risk for infection from HAT.
- We envisage this report will stimulate further HAT research in this old focus to estimate the prevalence and associated risk to the communities, game rangers, and tourists.
